# Longitudinal, Retrospective Use of a Circulating Tumor DNA Methylation Signature Successfully Captures Small Cell Evolution in a Patient With Metastatic EGFR-Mutant Non–Small Cell Lung Cancer

**DOI:** 10.1200/PO-25-00795

**Published:** 2026-04-03

**Authors:** Tejas Patil, Amy Guimarães-Young, Jill Tsai, Dara L. Aisner, Leslie Bucheit, Anton Valouev

**Affiliations:** ^1^ Division of Medical Oncology, Department of Medicine, University of Colorado School of Medicine, Aurora, CO; ^2^ Department of Pathology, University of Colorado Anschutz Medical Campus, Aurora, CO; ^3^ Guardant Health, Inc, Redwood City, CA

## Introduction

Small cell transformation (t-SCLC) in EGFR-mutant (EGFRm) non–small cell lung cancer (NSCLC) is a known mechanism of resistance to EGFR tyrosine kinase inhibitors, occurring in 3%-5% of patients.^1-5^ Transformation from adenocarcinoma to small cell lung cancer (SCLC) is characterized by high proliferative activity, expression of neuroendocrine markers, and biallelic loss of RB1 and p53 proteins through a combination of loss-of-function mutations and/or loss of heterozygosity.^1-9^ Pretreatment TP53 and RB1 mutations may predispose patients with EGFR mutations to subsequent small cell transformation.^8,9^ Clinically, t-SCLC has aggressive disease biology and is associated with poor clinical outcomes, with most patients receiving platinum-etoposide chemotherapy.^1-9^

Tissue biopsy is the gold standard for diagnosing t-SCLC.^4,10^ However, repeat biopsies can be limited by procedural risk, patient comorbidities, and tumor accessibility. Even when tissue biopsies are obtained, resistance alterations may be missed due to subclonality, limited tumor cellularity, or spatial heterogeneity across metastatic sites, resulting in incomplete characterization of tumor evolution from single-site tissue sampling.

Circulating tumor DNA (ctDNA) offers a minimally invasive approach for longitudinal tumor monitoring in EGFRm NSCLC. Although genomic ctDNA assays have been used to detect resistance mechanisms, including small cell transformation,^11,12^ the challenge is that small cell transformation is often accompanied by widespread epigenetic (non-CpG methylation) reprogramming within affected cells.^13^ Therefore, methylation-based ctDNA assays can provide complimentary information to genomic testing and improve detection of t-SCLC.^11-13^

Here, we describe a patient with EGFRm NSCLC who developed t-SCLC and demonstrate how longitudinal use of plasma-based methylation analysis successfully corresponded with small cell evolution throughout the disease course.

## Results

### Case

A 52-year-old woman with no smoking history presented with 3 weeks of progressive shoulder pain, dry cough, and dyspnea, and on examination was found to have decreased breath sounds along the right side with dullness to percussion up to the third intercostal space. Chest computed tomography (CT) imaging revealed extensive right-sided pleural thickening, pleural nodularity, and a large pleural effusion. A right-sided thoracentesis demonstrated atypical cells, suspicious for adenocarcinoma. Subsequent positron emission tomography (PET)/CT showed right-sided pleural-based disease (maximum standardized uptake value of 7.6 at the lung apex) along with mediastinal and precrural lymph node involvement. An brain MRI revealed no intracranial metastasis.

A core needle biopsy of the pleura revealed moderately differentiated adenocarcinoma with immunohistochemistry positive for cytokeratin 7 and thyroid transcription factor 1 and negative for cytokeratin 20, consistent with lung adenocarcinoma (LUAD). Tissue-based DNA and RNA next-generation sequencing (NGS) identified an *EGFR* exon 19 deletion (p.E746_A750del) and co-occurring pathogenic *CTNNB1* (p.S37F) and *TP53* (p.G245_N247del) alterations. Pretreatment ctDNA NGS (Guardant360 CDx) also identified the same somatic alterations, along with a pathogenic *PIK3CA* mutation (p.E545K; 0.1%; Table 1).

**TABLE 1.** Molecular Results at Associated Clinical Time Points

Time Point	Disease Course	Tissue NGS Results^a^	Guardant CDx^b^ ctDNA Results With Variant Allele Fraction (VAF %)	ctDNA MLS^c^ SCLC Binary Call	ctDNA MLS Deconvolution Proportions
0	Diagnosis (pretreatment)	*EGFR* c. 2236_2250del (p.E746_A750del) *TP53* c.733_741del (p.G245_N247del) *CTNNB1* c.110C>T (p.S37F) *PIK3CA* c.1633G>A (p.E545K) *Performed on pleural biopsy*	*EGFR* c. 2236_2250del (p.E746_A750del) (2.2%) *TP53* c.733_741del (p.G245_N247del) (2.0%) *CTNNB1* c.110C>T (p.S37F) (0.7%) *PIK3CA* c.1633G>A (p.E545K) (0.1%)	Not performed Insufficient material remaining	Not performed Insufficient material remaining
1	Radiographic progression on first-line osimertinib. t-SCLC identified *Samples obtained before treatment for t-SCLC*	*EGFR* c. 2236_2250del (p.E746_A750del) *TP53* c.733_741del (p.G245_N247del) ***RB1* c.1345G>A (p.G449R)** *PIK3CA* c.1633G>A (p.E545K) *PIK3CA* c.2176G>A (p.E726K) *Performed on soft tissue mass with small cell histology* *Note: Specimen underperformed as demonstrated by low coverage and other quality metrics*	*EGFR* c. 2236_2250del (p.E746_A750del) (25.8%) ***EGFR* c.2390G>C (p.C797S) (0.1%)** ***RB1* c.1345G>A (p.G449R) (11.6%)** *PIK3CA* c.1633G>A (p.E545K) (21.2%) *PIK3CA* c.2176G>A (p.E726K) (22.6%) *RIT1* c.241G>C (p.E81Q) (14.4%) *RIT1* c.270G>C (p.M90I) (14.5%) *BRCA2* copy number loss *EGFR* amplification (2+)^d^ *FGFR1* amplification (1+) *CCNE1* amplification (1+) *ESR1* amplification (1+) Not detected *TP53* c.733_741del (p.G245_N247del) (ND) *CTNNB1* c.110C>T (p.S37F) (ND)	Detected	LUAD: >90% LUSC: <10% SCLC: <10%
2	Radiographic progression 12 months after platinum etoposide for t-SCLC. *Samples collected while patient on osimertinib monotherapy*	*EGFR* c. 2236_2250del (p.E746_A750del) ***EGFR* c.2390G>C (p.C797S)** *CTNNB1* c.110C>T (p.S37F) *TP53* c.733_741del (p.G245_N247del) *RAD50* c.1387G>T (p.E463*) *Performed on axillary lymph node with adenocarcinoma histology*	*EGFR* c. 2236_2250del (p.E746_A750del) (0.5%) *PIK3CA* c.1633G>A (p.E545K) (0.5%) *RIT1* c.270G>C (p.M90I) (0.5%) *TP53* c.733_741del (p.G245_N247del) (1.2%) Not detected ***EGFR* c.2390G>C (p.C797S) (ND)** ***RB1* c.1345G>A (p.G449R) (ND)** *CTNNB1* c.110C>T (p.S37F) (ND) *PIK3CA* c.2176G>A (p.E726K) (ND) *RIT1* c.241G>C (p.E81Q) (ND) *EGFR* amplification (ND) *FGFR1* amplification (ND) *CCNE1* amplification (ND) *ESR1* amplification (ND)	Detected	LUAD: >90% LUSC: <10% SCLC: <10%
3	Radiographic oligoprogression in left and right upper lung nodules while on osimertinib with maintenance pemetrexed (s/p osimertinib, carboplatin, pemetrexed, and bevacizumab) *Sample collected before radiation therapy of oligoprogressive lesions*	*No biopsy obtained*	*EGFR* c. 2236_2250del (p.E746_A750del) (4.0%) *PIK3CA* c.1633G>A (p.E545K) (1.9%) *PIK3CA* c.2176G>A (p.E726K) (2.6%) *RIT1* c.270G>C (p.M90I) (1.8%) *TP53* c.733_741del (p.G245_N247del) (5.8%) Not detected ***EGFR* c.2390G>C (p.C797S) (ND)** ***RB1* c.1345G>A (p.G449R) (ND)** *CTNNB1* c.110C>T (p.S37F) (ND) *RIT1* c.241G>C (p.E81Q) (ND) *BRCA2* copy number loss (ND) *EGFR* amplification (ND) *FGFR1* amplification (ND) *CCNE1* amplification (ND) *ESR1* amplification (ND)	Detected	LUAD: 90% LUSC: <10% SCLC: 10%
4	Widespread retroperitoneal and axillary adenopathy detected on PET/CT while on osimertinib and maintenance pemetrexed *Samples collected before clinical trial enrollment*	*Biopsy used for clinical trial enrollment. No tissue-based molecular testing performed*	*EGFR* c. 2236_2250del (p.E746_A750del) (25.8%) ***EGFR* c.2390G>C (p.C797S) (0.1%)** ***RB1* c.1345G>A (p.G449R) (2.0%)** *PIK3CA* c.1633G>A (p.E545K) (5.3%) *PIK3CA* c.2176G>A (p.E726K) (3.9%) *RIT1* c.270G>C (p.M90I) (4.5%) *RIT1* c.241G>C (p.E81Q) (4.9%) Not detected *CTNNB1* c.110C>T (p.S37F) (ND) *TP53* c.733_741del (p.G245_N247del) (ND) *BRCA2* copy number loss (ND) *EGFR* amplification (ND) *FGFR1* amplification (ND) *CCNE1* amplification (ND) *ESR1* amplification (ND)	Detected	LUAD: 80% LUSC: <10% SCLC: 20%
5	Progression with new subcutaneous nodules while on amivantamab *Biopsy obtained before initiating osimertinib + paclitaxel*	*EGFR* c. 2236_2250del (p.E746_A750del) *TP53* c.733_741del (p.G245_N247del) ***RB1* c.1345G>A (p.G449R)** *PIK3CA* c.1633G>A (p.E545K) *PIK3CA* c.2176G>A (p.E726K) *Performed on subcutaneous nodule with small cell histology*^e^	*Not collected*	NA	NA

NOTE. The bolded mutations are the resistance mutations to osimertinib.

Abbreviations: CT, computed tomography; ctDNA, circulating tumor DNA; LUAD, lung adenocarcinoma; LUSC, lung squamous cell carcinoma; MLS, molecular lung subtyping; NA, not applicable; ND, not detected; NGS, next generation sequencing; s/p, status-post; SCLC, small cell lung cancer; t-SCLC, transformed small cell lung cancer; VUS, variants of unclear significance.

^a^ VUS are not shown for either tissue- or ctDNA-based testing. Neither the *RIT1* gene nor evaluation for copy number variations (eg, amplifications) is part of the tissue-based NGS assay used in this case report. MANE Select transcripts used for all reported alterations.

^b^ Guardant360 CDx is a 74-gene panel.

^c^ MLS established using the methylation data from the Guardant360 Liquid assay. Although >740 genes are part of the Guardant360 Liquid panel, only the epigenomic data were used in the evaluation of this patient's samples.

^d^ Gene amplification results in increased copies of the gene present in the cfDNA. The reported absolute copy number value represents the average copy number for the detected gene that was detected in circulating cfDNA. As the absolute number of copies in circulation is dependent on both tumor fraction and the magnitude of the tumor amplification, amplifications are reported on a semi-quantitative scale. For *CCNE1*, *EGFR*, *ESR1*, and *FGFR1*, three levels are reported: Low (+): Amplification magnitude is below the 50th percentile of amplifications detected by Guardant360. Medium (++): Amplification magnitude is between the 50th and 90th percentiles. High (+++): Amplification magnitude is above the 90th percentile. For *BRAF*, *CCND1*, *CCND2*, *CDK4*, *CDK6*, *ERBB2*, *FGFR2*, *KIT*, *KRAS*, *MET*, *PDGFRA*, *RAF1*, *MYC*, *PIK3CA*, and *AR*, two levels are reported: Medium (++): Amplification magnitude is below the 50th percentile of amplifications detected by Guardant360. High (+++): Amplification magnitude is above the 50th percentile.

^e^ NGS analysis performed retrospectively for the purposes of this case report.

**FIG 1.**
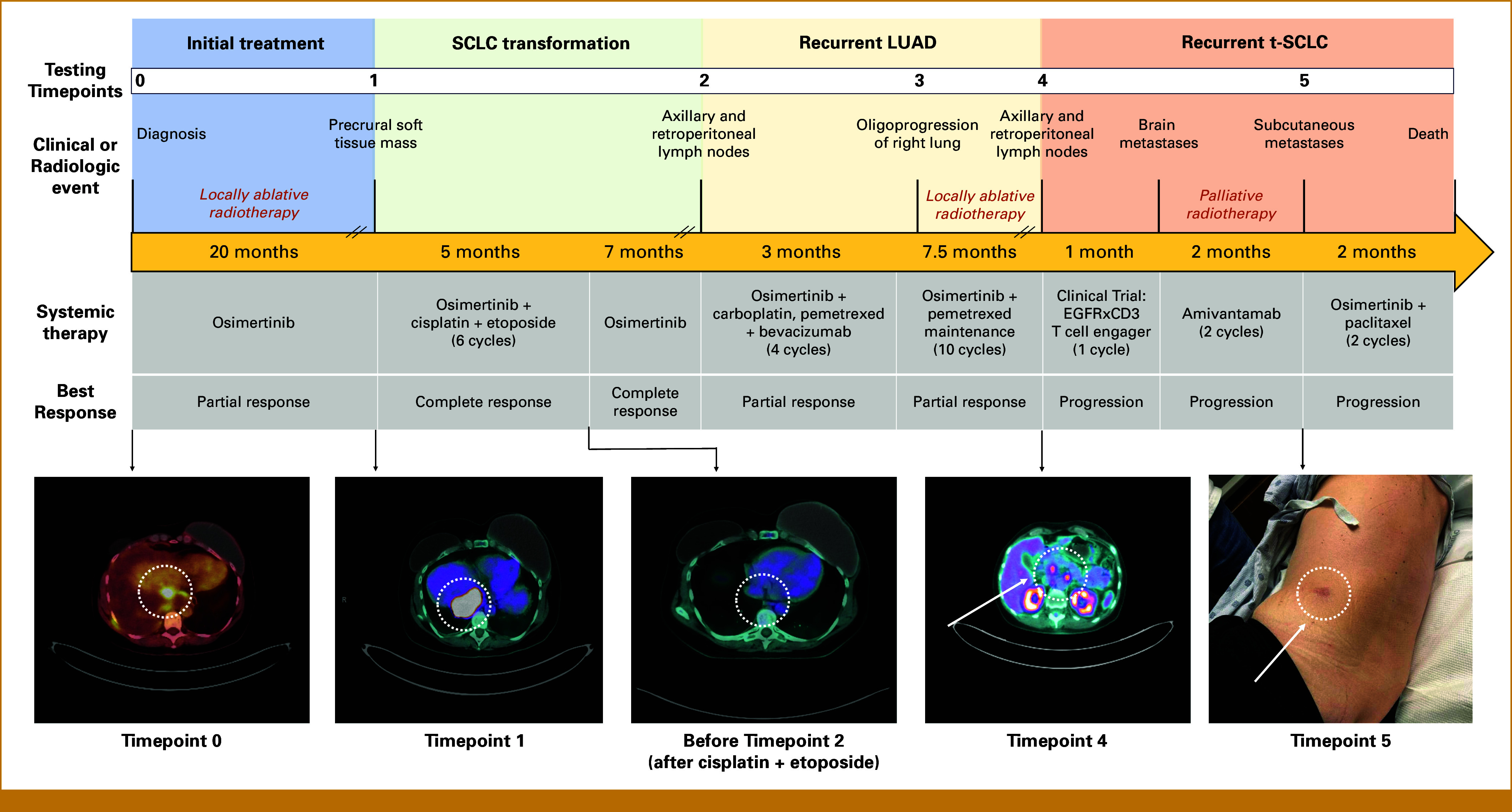
Schematic of patient's clinical course. Testing time points designate timing of ctDNA and/or tissue collected for molecular analyses. Hashmarks along yellow arrow indicate longer time frames not schematically represented to scale with shorter time intervals. Radiotherapy is represented above the pharmacologic agent patient was receiving at the time of procedure. IMRT was used to target the intra-abdominal lymph node (first instance) and sites of right upper and lower lung (second instance), whereas SRS was used to target six metastatic brain lesions. Best response was determined via radiologic evaluation of tumor. PET images at bottom of the figure demonstrate disease burden at each time point. ctDNA, circulating tumor DNA; IMRT, intensity-modulated radiation therapy; LUAD, lung adenocarcinoma; PET, positron emission tomography; SCLC, small cell lung cancer; t-SCLC, transformed small cell lung cancer.

**FIG 2.**
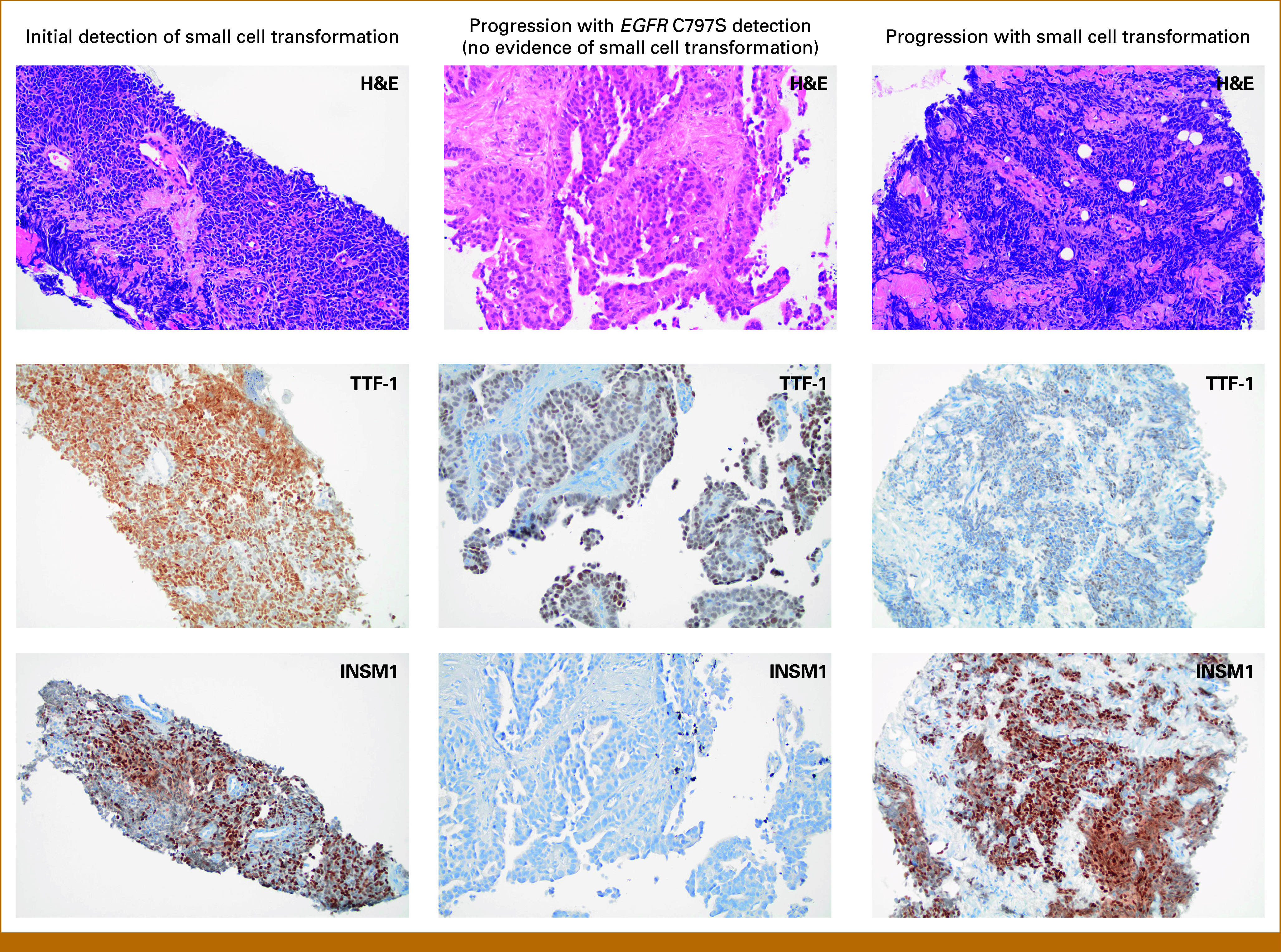
Histopathologic images of the patient's tumor at associated clinical time points. A precrural soft tissue mass biopsied 20 months into treatment with osimertinib (time point 1) demonstrating infiltrative small cells with minimal cytoplasm, nuclear molding, and smudgy, fine chromatin (H&E) positive for TTF-1 (a marker of lung origin) and INSM1 (a marker of neuroendocrine differentiation), consistent with t-SCLC (left column). Right axillary lymph node biopsied 7 months after completion of platinum etoposide chemotherapy while on osimertinib monotherapy (time point 2) demonstrating haphazardly arranged gland-forming cells of irregular/variable size (H&E) positive for TTF-1 and negative for INSM1, consistent with recurrent metastatic lung adenocarcinoma (center column). Subcutaneous nodule biopsied upon progression on amivantamab and osimertinib (time point 5) demonstrating recurrent t-SCLC positive for TTF-1 and INSM1 (right column). H&E, hematoxylin and eosin; ISNM1, insulinoma-associated protein 1; t-SCLC, transformed small cell lung cancer; TTF-1, thyroid transcription factor 1.

The patient received first-line osimertinib 80 mg once daily with rapid symptomatic improvement and reduction in her right-sided pleural disease. After 20 months on osimertinib, she developed a rapidly enlarging precrural soft tissue mass (Fig 1). A ctDNA sample at progression identified the known *EGFR* exon 19 deletion (p.E746_A750del; 25.8%), an on-target *EGFR* resistance mutation (p.C797S; 0.1%), and emergence of a pathogenic *RB1* mutation (p.G449R; 11.6%). A biopsy of the progressing lesion demonstrated small cell carcinoma, confirming t-SCLC (Fig 2). Tissue-based NGS identified the original *EGFR* exon 19 deletion along with co-occurring *TP53* (p.G245_N247del) and *RB1* (p.G449R) alterations.

The patient was treated with six cycles of cisplatin 75 mg/m^2^ with etoposide 100 mg/m^2^ D1-D3 every 21 days while continuing osimertinib 80 mg once daily. She achieved a complete radiographic response and remained on osimertinib 80 mg once daily after chemotherapy was complete. One year after the detection of t-SCLC, CT imaging showed new axillary and retroperitoneal lymphadenopathy. A right axillary lymph node biopsy revealed recurrent adenocarcinoma without t-SCLC features (Fig 2). Both tissue and plasma NGS revealed the original *EGFR* exon 19 deletion, although only the tissue NGS detected the prior *EGFR* C797S alteration (Table 1). Crucially, the prior *RB1* mutation (p.G449R) was not detected in either the tissue or ctDNA NGS results.

She remained on osimertinib 80 mg orally daily and received carboplatin AUC 5, pemetrexed 500 mg/m^2^, and bevacizumab 15 mg/kg every 21 days for four cycles. She had a partial radiographic response. After 10 cycles of maintenance pemetrexed, a PET/CT revealed worsening right axillary and retroperitoneal lymph nodes. A repeat axillary lymph node biopsy showed adenocarcinoma without t-SCLC features. Tissue-based molecular testing was not performed to preserve the specimen for clinical trial enrollment; however, ctDNA testing demonstrated re-emergence of the *RB1* mutation (p.G449R; 2.0%), along with known *EGFR* exon 19 deletion (p.E746_A750del; 7.1%) and *EGFR* (p.C797S; 0.2%). Despite multiple subsequent therapies, including a clinical trial with an EGFR-CD3 bispecific T-cell engager (ClinicalTrials.gov identifier: NCT04844073), amivantamab, and paclitaxel, she ultimately developed recurrent t-SCLC (as determined by a biopsy of rapidly progressive subcutaneous nodules) and died from progressive disease.

### Retrospective Methylation Analysis of ctDNA to Provide a Molecular Lung Subtype Prediction

All available on-treatment ctDNA samples underwent retrospective methylation analysis to generate a Molecular Lung Subtype (MLS) prediction. This classifier estimates proportional contributions of LUAD, squamous cell carcinoma, and small cell carcinoma based on methylation patterns. Although the patient's pretreatment ctDNA was unavailable for MLS analysis due to insufficient plasma quality (time point 0/baseline), all subsequent on-treatment samples were available for MLS assessment.

**FIG 3.**
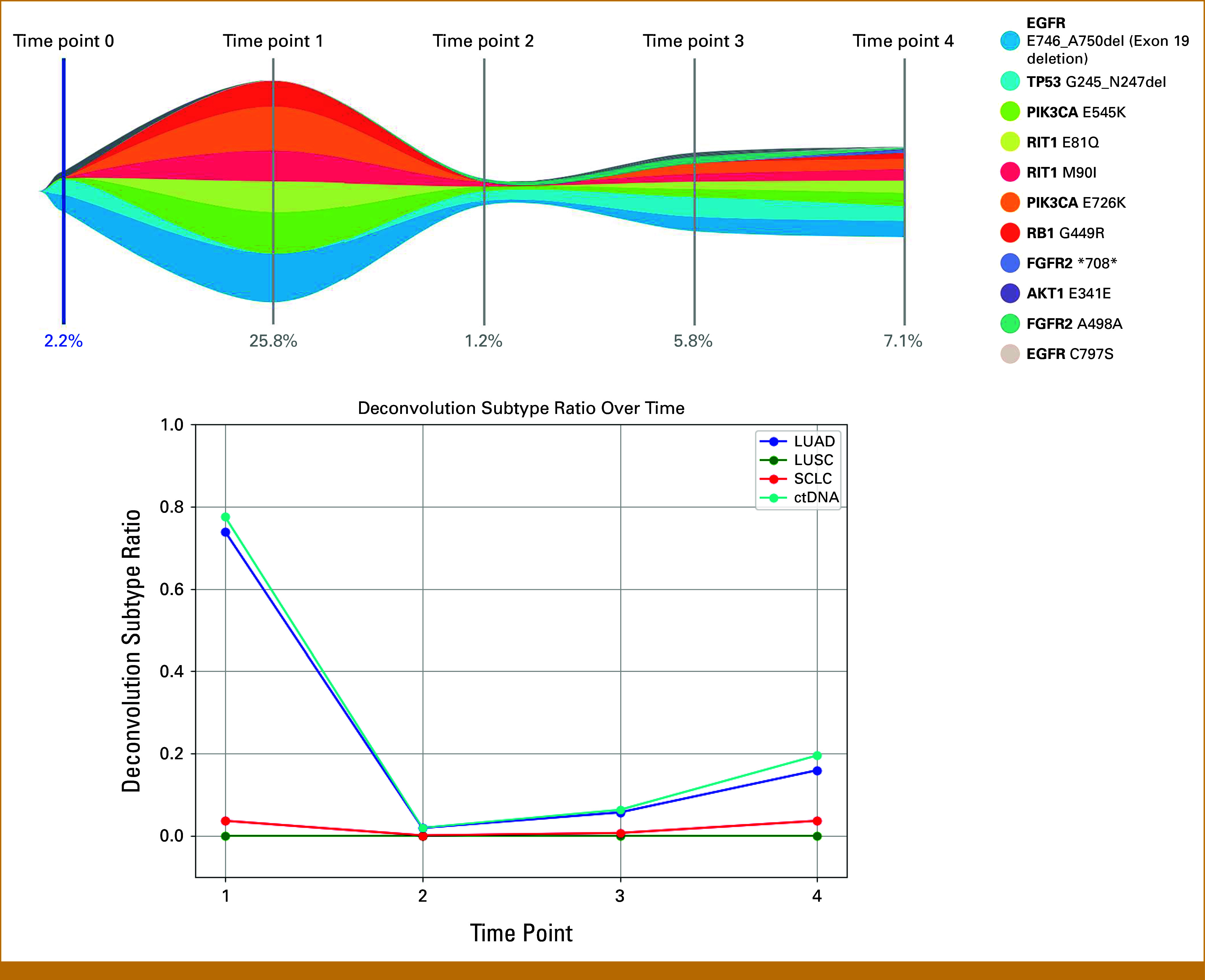
ctDNA monitoring and methylation-based deconvolution subtype ratio over time at on-treatment time points. Guardant 360 circulating tumor DNA response map showing dynamic changes in variant allele fractions across multiple time points in this patient's cancer journey (top). The corresponding deconvolution subtype ratio at matching time points is shown below. The MLSP is a deconvolution model used to identify the proportional contributions of LUAD, LUSC, and SCLC from a plasma sample using patient-specific subtype hypermethylation signature and noncancer component signature across regions. ctDNA, circulating tumor DNA; LUAD, lung adenocarcinoma; LUSC, lung squamous cell carcinoma; MLSP, molecular lung subtyping profile; SCLC, small cell lung cancer.

**FIG A1.**
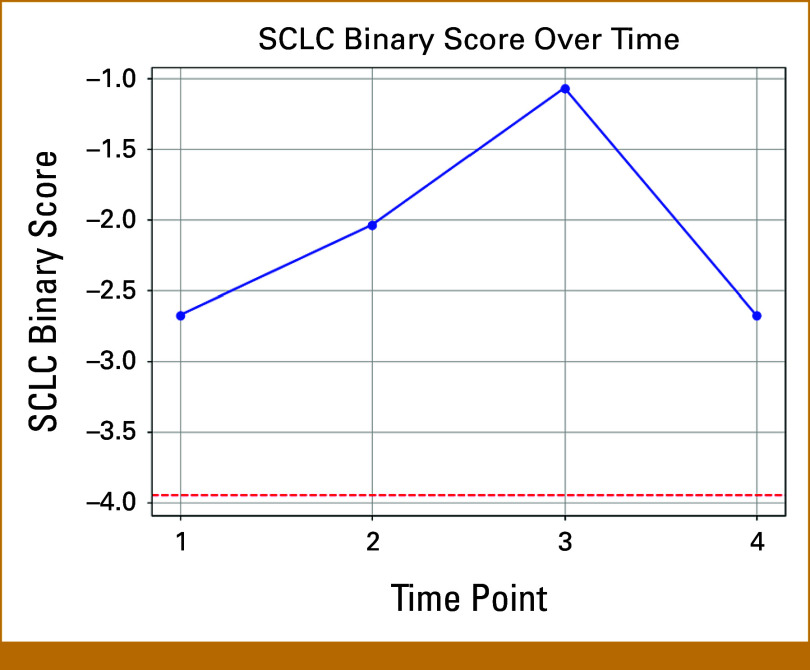
Small cell lung cancer binary score at associated time points. The MLSP is a deconvolution model used to identify the proportional contributions of LUAD, LUSC, and SCLC from a plasma sample. The threshold for calling SCLC in a plasma sample is shown with the dotted red line. The blue lines represent the binary SCLC score at each time point in the case report indicating that each plasma sample met the threshold to identify SCLC clones using the binary SCLC classifier. LUAD, lung adenocarcinoma; LUSC, lung squamous cell carcinoma; MLSP, molecular lung subtyping profile; SCLC, small cell lung cancer.

At the first evaluable time point, ctDNA demonstrated a mixed adenocarcinoma and SCLC methylation signature, concordant with clinical ctDNA NGS and tissue-confirmed t-SCLC (Fig 3; Appendix Fig A1). This dual subtype signal persisted across all subsequent time points, with changes in adenocarcinoma and SCLC proportions broadly paralleling clinical response and progression (Fig 3, Appendix Fig A1). Notably, MLS proportions did not correlate linearly with individual mutation variant allele frequencies (VAF), as determined by plasma-based NGS. For example, at time point 1, ctDNA NGS identified a pathogenic *RB1* at mutation (p.G449R) with a VAF of 11.6% and an SCLC proportion <10%, whereas at time point 3, *RB1* (p.G449R) was not detected although the calculated SCLC proportion was 10% (Table 1). These findings underscore the limitations of relying on single genomic alterations to infer small cell evolution and highlight the complementary nature of epigenomic profiling.

## Methods

### Patient

The patient provided written informed consent for all procedures, tests, treatments, and clinical trial participation under an institutional review board–approved protocol (COMIRB 09-0143).

### Testing

Diagnostic tissue underwent DNA/RNA NGS testing using STRATA (Ann Arbor, MI). Subsequent tissue sequencing was performed using a validated 498-gene hybrid capture assay (IDTxGen) at the Colorado Molecular Correlates Laboratory. Libraries were sequenced on the Illumina platform, and the data were processed using a customized analysis pipeline. The presumed amino acid changes derived from mutational testing are provided in the body of the manuscript (Table 1).

All ctDNA genomic analyses were conducted using the Guardant360 CDx assay.^14,15^ A retrospective methylation analysis was conducted using the Guardant Infinity platform, with epigenomic data used to derive an MLS predictor. The MLS predictor is a deconvolution model used to identify the proportional contributions of LUAD, lung squamous cell carcinoma, and SCLC from plasma. MLS prediction was generated using a validated deconvolution model assessing methylation across >9,000 cancer-associated regions, with reported analytic performance previously described.^16^

## Discussion

This case demonstrates the longitudinal integration of tissue-based and plasma-based molecular profiling in a patient with EGFRm NSCLC experiencing both on-target EGFR resistance and small cell transformation. To our knowledge, it is the first detailed clinical report showing serial plasma-based methylation analysis to track t-SCLC evolution alongside standard genomic testing. Although the use of epigenomic assays to noninvasively detect t-SCLC has been described,^13^ this case showcases how longitudinal use of epigenomic assays can accurately discriminate between two competing EGFRm NSCLC–resistant subclones and their response to anticancer therapies.

Current ctDNA assays primarily detect genomic alterations with limited sensitivity for identifying small cell transformation, which is largely driven by epigenomic reprogramming.^3-6,10-13^ Although *TP53* and *RB1* alterations are associated with an increased risk of small cell transformation in EGFRm NSCLC,^7-9^ as identified in this patient, they are neither necessary nor sufficient for its detection, particularly given the subclonal nature of acquired resistance and the epigenomic changes seen in t-SCLC. Plasma-based methylation profiling offers several potential advantages: minimal invasiveness, rapid turnaround, and the ability to capture tumor heterogeneity across metastatic sites. Deconvolution of subtype-specific methylation signals may also enable simultaneous monitoring of competing resistance clones, as illustrated here.

An exploratory analysis of SWOG S1403 (ClinicalTrials.gov identifier: NCT02438722) found clearance of EGFR ctDNA levels at 8 weeks from baseline to be predictive of progression-free-survival (PFS; hazard ratio [HR], 0.23) and overall survival (OS; HR, 0.44).^17^ Given the epigenomic changes associated with t-SCLC, there remains an unmet need for plasma-based assays that can identify and track epigenomic-driven resistance. Here we show that clearance of plasma-based SCLC methylation signatures correlated with treatment response to cisplatin + etoposide in t-SCLC. This is relevant given the poor prognosis of patients with t-SCLC with a median PFS of 3.4 months (with platinum-etoposide) and 2.7 months (with taxanes).^1^ Efficacy to immune checkpoint inhibitors is also minimal,^18^ suggesting that t-SCLC has a different immunologic profile relative to smoking-associated SCLC. Although tarlatamab, a bispecific delta-like ligand 3 (DLL3)–directed T-cell engager, has demonstrated improved OS compared with chemotherapy in patients with nontransformed relapsed SCLC (HR, 0.60),^19^ it remains unclear whether a similar benefit extends to EGFRm NSCLC with small cell transformation. In a recent study, the median duration of tarlatamab treatment in this population was only 1.45 months, with higher response rates observed among patients with DLL3 expression exceeding 80%.^20^

This report has limitations. There was no baseline plasma specimen available for MLS evaluation due to insufficient plasma quality, limiting our ability to delineate the transition from adenocarcinoma to t-SCLC. Although retrospective MLS assessments did correlate with treatment changes, clinical decisions were ultimately made using plasma genomic and tissue samples. Prospective studies are needed to define how MLS proportions correlate with tumor burden, treatment response, and outcomes.

In conclusion, this case highlights the utility of longitudinal plasma-based monitoring in metastatic EGFR-mutant NSCLC, enabling discrimination of acquired resistance clones. Blood-based molecular lung subtyping retrospectively aligned with tissue-confirmed small cell transformation, supporting the feasibility of methylation-based assays to detect and monitor t-SCLC. Prospective studies are needed to define their clinical utility and integration into routine care.

## Data Availability

A data sharing statement provided by the authors is available with this article at DOI https://doi.org/10.1200/PO-25-00795. All the data and resources generated for this study are available in the article or from the corresponding author upon request.
